# Meal Pattern in the Colombian Population: Results of the National Nutrition Survey. ENSIN, 2015

**DOI:** 10.1155/2022/1047524

**Published:** 2022-08-25

**Authors:** María del Pilar Zea, Oscar F. Herrán

**Affiliations:** ^1^Pontificia Universidad Javeriana Seccional Cali, Facultad de Ciencias de la Salud, Departamento de Alimentación y Nutrición, Calle 18 N 118-250, Cali, Valle del Cauca, Colombia; ^2^Escuela de Nutrición y Dietética, Universidad Industrial de Santander, Carrera 32 No. 29-31, Bucaramanga, Santander, Colombia

## Abstract

**Background:**

Information on meal patterns (type, number, relative contribution to energy/day (%), time, and location of meals) is limited or nonexistent.

**Design:**

Cross-sectional, nationally representative surveys. *Setting.* Colombia. *Participants*. *n* = 26,115 from 3 to 64 years old. The sample analyzed included 3,127 children between 3 and 4 years old, 13,384 children between 5 and 17 years old, and 9,604 adults between 18 and 64 years old. *Data Analysis.* Meal patterns were described by age group. Through multiple linear regression, crude and adjusted differences in the categories of the covariates studied were estimated. The number/day of meals was the dependent variable.

**Results:**

The number of meals/day (mean ± SD) was 4.4 ± 0.0, without differences by sex (*P* = 0.068), current weight (*P* = 0.336) , or wealth index (*P* = 0.480), but there were differences in the level of education of the head of the household (*P* < 0.0001) and the level of food security of the household (*P* < 0.0001). A total of 96.8% of the population eats 3 or more meals/day (95% CI: 96.2, 97.2). The consumption frequency (mean ± SD) of the three main meals was 0.95 ± 0.0 “times/day,” 1.0 ± 0.0 and 0.95 ± 0.0, for breakfast, lunch, and dinner, respectively. Lunch is the meal that makes the greatest relative contribution to the total energy consumed (energy/day), 33.9% (95% CI: 32.7, 35.1). Breakfast is eaten outside the home by 13.0% of the subjects, lunch by 26.0%, and dinner by 3.8%. The minimum fasting interval is 9 hours and the maximum is 10 hours and 30 minutes. The meal pattern is equivalent to type “A,” with three main meals and two or three intermediate meals (midmorning and midafternoon) taken during 15 hours of the day.

**Conclusions:**

All age groups had more than four meals/day. The number is directly related to socioeconomic level. Lunch is the main meal.

## 1. Introduction

The dietary transition as a complementary concept to the nutritional transition is based on the establishment of dietary patterns and on the study of the dietary changes that occur in such patterns [1–4]. It is important to identify dietary consumption patterns (Traditional, Western, Prudent, etc.), as well as the type and number of the meals that compose them (meal patterns or eating patterns) [5–7]. It is possible to establish a pattern of meals and associate it with cultural, political, social, economic, and biological changes [5, 8–11]. Meal patterns are also associated with the time in which they are made (meal timing) and with the quality of the diet [5, 12–14]. A key aspect is to establish the definition of what the term meal means. There are many definitions, but four are the most relevant: the one based on the time of the day when it is performed, the one based on the quantity and quality of the foods that compose it, and the one, that is, self-reported by the subjects and/or based on a pre elaborated list and another one called neutral, which presupposes a post classification by convenience by the researcher in order to establish comparisons [5, 15]. The used definition influences the results by relating the pattern of meals with the consumption of nutrients, the quality of the diet, the frequency of consumption, or even health-disease events [5]. The main sources of data to establish a meal pattern are the reminders of the dietary consumption of the last 24 hours (24-hour dietary recall, (24 HR)) and the food frequency questionnaires (FFQ) [5, 16].

In Colombia, based on the two most recent National Surveys of the Nutritional Situation (ENSIN), which were conducted in 2010 and 2015, three dietary patterns were identified and the stages of alimentary and nutritional transitions were established [2, 17]. In addition, adherence to dietary consumption patterns by geographic area [4], consumption of macro and micronutrients, their dietary sources, and the quality of the diet throughout the life cycle was characterized, all associated with biological and socioeconomic characteristics [18, 19]. In Colombia, as in many countries, the formal study of meal patterns is limited. The study of meal patterns is a developing field, and the type of food, its frequency of consumption [20], the number of meals/day, and the time in which they are made have been found to be associated with circadian rhythm [12, 13], metabolic changes [21], body weight and its control [22, 23], mediating events of chronic disease such as high levels of cholesterol and hypertension, and the development of diabetes and cardiovascular disease [12–34]. Meal patterns are also associated with glycemic alterations in pregnant women and preterm births [35].

Meal patterns can function as a potential cause and consequence. As a cause, they are linked to biological and health-disease events. Complementarily, they are the consequence of complex interactions between culture, economic and political crisis, climate change, food marketing, employment, income, migrations, educational level, and food insecurity; additionally, in Colombia, they are the consequence of internal conflict, violence and forced internal displacement of populations that are associated with territorial control by violent groups to develop illegal economies [36–38].

Based on information collected in 2015 in Colombia by the National Nutritional Situation Survey (ENSIN-2015) [39, 40], the objectives of this study were: (*a)* to estimate the number of meals/day associated with some biological and socioeconomic variables of interest, (*b)* to estimate crude and adjusted differences in the number of meals in the categories of these variables, (*c)* to estimate the absolute kilocalories and relative (%) contributions of each type of meal to the total energy intake/day (kilocalories), (*d*) to establish the prevalence of meal intake (%), the frequency of consumption (times/day) and the place where the different types of meal are made, and (*e*) to estimate and describe the time at which they are taken.

## 2. Methods

Colombia is a developing country with medium income and widespread social inequalities, that is, located in the northeastern corner of South America [41, 42]. In Colombia during 2015, under the leadership of the Colombian Institute of Family Welfare (ICBF) and the Ministry of Health (MinSalud), ENSIN-2015 was conducted. In ENSIN-2015, 44,202 households were surveyed, representing 4,739 groups of 295 strata representing 99% of the country's population. The methods, the populations studied, and the scope and limitations of ENSIN-2015 have already been published [39]. Minor children between 3 and 17 years old and adults between 18 and 64 years old, excluding pregnant women and girls, were the target population of this analysis. The study population answered, among others, a FFQ, a 24 HR, and a sociodemographic survey. The ENSIN-2015 included 151,343 people, of which 28,902 answered questions about the type of food consumed (yes/no), the frequency, and the place, where they were made. After excluding pregnant women (n = 1,939) and subjects outside the age range or with incomplete information (n = 848), the final sample comprised 26,115 subjects including 3,127 children between 3 and 4 years of age, 13,384 children between 5 and 17 years of age, and 9,604 adults between 18 and 64 years of age.

### 2.1. Data Sources

Based on the FFQ, we established the number of meals/day, the prevalence (%), the frequency of consumption (times/day) of each meal, and the place, where it was consumed (not where they were prepared). Based on the 24 HR, we established the time of consumption of each type of meal (Hour:Minutes), the usual intake/day (kilocalories), and the relative contribution (%) of each meal to the total intake.

The main outcome variables were: (*a*) the number of meals per day (meals/day), (*b*) the prevalence (%) of each type of meal, (*c*) the frequency of each meal (times/day), (*d*) the predominant place (%) where they are taken, and (*e*) the time of day at which they are taken (Median, P_25_, and P_75_). Based on the FFQ, the number of meals was estimated by investigating whether eight specific meals from a preestablished list were usually made (before breakfast, breakfast, mid-morning, lunch, mid-afternoon, dinner, after dinner, and another) and the prevalence of realization (%) through a dichotomous response (yes/no) on the usual intake in the last week. The frequency of each type of meal was established based on five categories referring to the past week (every day, between five and six days, between three and four days, between two and three days, and between one and two days). After translating the previous response options to a continuous time variable, the frequency of each type of meal was expressed as times per day (times/day). In addition, the FFQ inquired about the predominant location of each type of meal, also referring to the last week. For this variable, there were ten response options (home, school/university, family or friend's house, restaurant, cafeteria, street-mobile stand, institutional program, work, transportation, and others). Based on 24 HR, the average energy (kilocalories) provided by each type of meal and the relative contribution (%) that each of them makes to the total energy consumed per day (kilocalories/day) were estimated. ENSIN-2015 applied a repeated 24 HR in subsample [39], following the methodology developed in 1999 by the United States Department of Agriculture (USDA) [43, 44]. The 24 HR had a response rate of 92%. The distribution of the intake of kilocalories/day was normalized and corrected by incorporating the intraindividual variability with the methods proposed by the University of Iowa and the PC-Side software, v1.0 [45]; therefore, the reported intake (kilocalories) and the relative contribution (%) correspond to the usual intake of the subjects. In summary, the usual intake is a value derived from the two 24 HR (but not as a simple average), to represent the long-term intake, rather than the intake of the last 24 hours [45].

In addition, based on 24 HR, the time at which each type of meal was eaten was estimated (Median, P_25_, and P_75_).

Eight biological and sociodemographic variables were the covariates of interest; these covariates, along with the FFQ and the 24-hour-recall (24 HR), were assessed by direct interviews by dietitians, nutritionists, and trained personnel. Household food insecurity was established with the Latin American and Caribbean Scale (ELCSA) adopted by the Food and Agriculture Organization of the United Nations (FAO) [46]. The ELCSA classifies households as safe and unsafe and the level of food insecurity as mild, moderate, or severe [46]. The wealth index is a continuous indicator of the socioeconomic level of the household, and it was categorized into quartiles. It was determined in the total sample of the survey (ENSIN-2015). It was established based on the analysis of the main components of a set of physical characteristics of the household, the goods in the household, and the availability of services, all of which were previously established in international surveys of demography and health [39, 47]. The level of education of the head of the household was established based on the approved years of study. The geographical region is a variable that represents the territory and the conditions of structural, economic, and cultural development of the subjects. Colombia has five geographical regions [39]. Bogotá is the capital of the country and, together with the central region, has the largest human development index, while the Pacific region along with the Amazonia-Orinoquia has the worst human development index [42]. The most prevalent population in the Pacific region is Afro-descendant individuals, followed by indigenous and mestizo individuals. The level of urbanism was established based on the concentration of the population in three categories: urban centers have more than one million inhabitants and large cities; small towns have between one hundred thousand and one million inhabitants; dispersed populations have fewer than one hundred thousand inhabitants [39].

Anthropometric measurements were obtained from all household members with the use of calibrated standards, techniques, and instruments. In ENSIN-2015, height was measured with the use of stadiometers (ShorrBoard), with a sensitivity of 1 mm. Weight was measured with SECA 874 scales, with a precision of 100 g [39]. In minors, *Z*-scores were established for the height/age and Body Mass Index (BMI) indicators according to the growth standards of the World Health Organization (WHO) [48].

### 2.2. Data Analysis

All analyses were conducted under the consideration of the complex sampling design and using Stata v14.1 [49] to, (*a*) estimate and describe the average number of meals/day by the categories of the covariates of interest, (*b*) estimate the crude and adjusted differences in the number of meals/day between categories of the covariates studied and by age group, (*c*) describe the prevalence (%) of the type of meal made by age, the frequency/day (times/day), the usual energy provided by each meal (kilocalories) and the relative contribution of each meal (%) to the total energy consumed in the day (kilocalories/day), (d) establish the prevalence (%) of the place where each type of meal is made by age group, and (*e*) establish the time (Median, P_25_, P_50_) at which each meal was taken. The variables were represented with means or proportions ± standard error (SE) and 95% confidence intervals (95% CI). The adjusted differences were obtained through multiple linear regression models with number of meals as a continuous result and biological and sociodemographic variables as predictors except the height-for-age or height- and BMI-for-age or BMI. The estimates for education come from a model that excludes the wealth index and food security, which could be on the causal path. The wealth index estimates excluded food security.

The study was conducted according to the guidelines laid down in the Declaration of Helsinki [50]. Consent for participation in the survey was obtained by the Colombian Institute of Family Welfare prior to enrollment [39]. The ethics committee in health research of the Universidad Industrial de Santander determined that analyses of these anonymized data were exempt from review.

## 3. Results

A total of 49.4% of the participants were men. A total of 8.2% of the children presented stunting. A total of 13.6% (95% CI: 11.0, 16.8) of children between 3 and 4 years of age, 15% (95% CI: 13.2, 16.9) of children between 5 and 17 years of age, and 14.5% (95% CI: 12.8, 16.4) of adults were obese (*Z*-score >2 or BMI ≥30). A total of 24.7% (95% CI: 22.4, 27.2) of adults had primary education or less, and 9% (95% CI: 6.9, 11.6) had higher education or more. 38.0% (95% CI: 29.5, 49.3) of the participants were in *Q*_1_ of the wealth index, and 13.7% (95% CI: 10.8, 17.1) were in *Q*_4_. 36% (95% CI: 26.6, 46.5) live in the four main cities of the country. The number of meals/day (mean ± SD) in the population aged 3 to 64 years was 4.4 ± 0.0; and there were no differences based on sex, *P* value = 0.068, current weight, *P* value = 0.336 or wealth index *P* value = 0.480, but there were differences based on the level of education of the head of the household, *P* value < 0.0001 and the household food security, *P* value < 0.0001. The number of meals/day in households with heads with less than primary school was 4.3 ± 0.0, 0.4 meals/day less than in households, where the head has a complete university education, 4.7 ± 0.1, for trend *P-*value<0.0001.

In Tables 1–4 show the mean number of meals/day and the crude and adjusted differences between the categories of the socioeconomic variables studied. There are significant differences in favor of men, and the relationship between the number of meals/day with the level of education, height-for-age (*Z*-score), and household food security is direct. In addition to the above, an inverse relationship between the number of meals/day and body mass index is observed in adults.  Figure 9(s) shows the mean number of meals/day by age.

The point prevalence of having one or fewer meals/day was 0.00% (95% CI: 0.00, 0.00), that of having two meals was 0.03% (95% CI: 0.03, 0.04), that of having three meals/day was 0.23% (95% CI: 0.22, 0.25), that of having four meals/day was 0.27% (95% CI: 0.25, 0.28), that of having five meals/day was 0.28% (95% CI: 0.27, 0.29), that of having six meals/day was 0.14% (95% CI: 0.13, 0.15), and that of having seven meals was 0.04% (95% CI: 0.04, 0.05). Finally, the prevalence of eating eight meals/day was 0.00% (95% CI: 0.00, 0.00). A total of 96.8% of the population had 3 or more meals/day (95% CI: 96.2, 97.2). Figure 2(s) shows the probability of having a specific type of meal/day according to the level of food security of the household.

The prevalence of before breakfast was 25.0% (95% CI: 22.8, 27.3), that of breakfast was 95.6% (95% CI: 94.9, 96.2), that of the midmorning was 46.8% (95% CI: 44.7, 48.9), the lunch was 98.7% (95% CI: 98.1, 99.0), the midafternoon was 51.9% (95% CI: 50.0, 53.7), the dinner was 94.8% (95% CI: 93.7, 95.6), and finally, the prevalence of after dinner was 25.3% (95% CI: 23.2, 27.5). The frequency of consumption, times/day (mean ± SD) of the three main meals was 0.95 ± 0.0, 1.0 ± 0.0, and 0.95 ± 0.0 for breakfast, lunch, and dinner, respectively. Lunch was the meal that made the greatest relative contribution to the total energy consumed (energy/day), 33.9% (95% CI: 32.7, 35.1), followed by dinner, with a relative contribution of 24.7% (95% CI: 23.8, 25.6), and breakfast, with a relative contribution of 22.7% (95% CI: 21.4, 23.9).  Table 5 presents the prevalence and frequency (times/day) by type of meal, in addition to the absolute kilocalories and relative (%) contribution to the total energy/day by the age groups studied. The relative contribution (%) that lunch makes to the total energy/day is directly related to the level of education of the head of the household, *P* value = 0.012. The relationship between the relative contribution (%) and the wealth index is direct with the midmorning, *P* value = 0.001, lunch, *P*=0.045, the midafternoon, *P*=0.010 and with after-dinner *P* value 0.020.  Figure 3(s) shows the relative contribution (%) of each type of food to the total energy/day according to age.

All meals were mostly eaten at home; breakfast was done outside the home by 13.0% of the subjects, lunch by 26.0%, and dinner by 3.8%.  Table 6 presents in detail the place where each type of meal is made by the age groups studied. Finally, the time (Hour:Minutes) at which each meal is done (Median, P_25_, P_75_) is 6:30 (6:00, 7:20) before breakfast, 8:00 (7:00, 9:00) for breakfast, 10:00 (9:30, 11:00) for midmorning, 12:30 (12:00, 13:00) for lunch, 16:00 (15:00, 17:00) for midafternoon, 19:00 (18:00, 19:30) for dinner and 20:20 (19:30, 21:00) for after dinner. Figure 4(s) shows, in addition to the time of completion, the relative density by the number of food records at each meal time. Lunch is the most concentrated meal regarding the time in which it is eaten, and breakfast and midafternoon are the most dispersed at the time of eating.

## 4. Discussion

The findings of this study established the pattern of meals in the Colombian population between 3 and 64 years of age. In 2015, all age groups had more than four meals/day. The number is directly related to variables that represent socioeconomic level (wealth index) or proximal to it (household food security and level of education). The home continues to be the main place, where all meals are made. Lunch and midafternoon are the two meals that are most often eaten outside the home. The lower prevalence of midmorning and midafternoon is what differentiates insecure households from those with food security. Contrary to popular belief in this population, breakfast is not the main meal in terms of absolute kilocalories or relative (%) contribution of energy/day. Lunch is the main meal, with an advantage of nine percentage points in the relative contribution over dinner and breakfast. According to the time in which the meals are made, lunch is the most concentrated, and breakfast and midafternoon are the most dispersed during the day. The meal pattern reported in this study is classified as type A [13]. Since there is no reason to suppose that this pattern changed in the last decade in Colombia, it is reasonable to state that it is a potential risk factor for the development of chronic diseases. The “A” meal pattern is a common meal pattern in which the obesity epidemic developed and in which diabetes and associated chronic diseases have emerged [13].

As already noted, the definition and classification of the term “meal” influence subsequent results [5], which makes it difficult to compare our findings. A study with noninstitutionalized adults in the USA (>45 y), where the three main meals were investigated and the rest were called “snacks” showed that at least 67% ate three or more meals/day (herein, the prevalence was 96.8%), and dinner had the highest prevalence (36%) [6]. A study conducted among U.S Adventists (≥30 y) that measured meals with six categories found that they eat 4 ± 1 meals/day (mean ± SD) and that lunch is the meal with the least variability in the time of day it is eaten [30]. A study of Puerto Rican children aged 10–17 years, in which eight types of meals were established, showed that they consumed between 4.3 ± 1.4 and 5.0 ± 1.1 meals/day (mean ± SD). Younger children had more meals/day and those with inadequate weight less meals/day [16]. The report of fewer meals with a higher body mass index (BMI) in Puerto Rican children, as also reported in this study, is likely to be a reporting bias, which is well known when studying dietary intake [51]. A study in Swedish women (>45 y), where the number of meals was established based on the time of day at which they were made, showed that they consumed between 6.1 vs. 5.2 meals/day, with an advantage for obese women compared to those who had adequate weight. In addition, it was established that obese women ate more meals/day in the evening (after 16:00 hours) than women with adequate weight [24]. In adults residing in the USA (20–70 y), based on eight preestablished meal times, it was estimated that they ate an average of 3.9 ± 0.8 meals/day. In addition, the percentage of meals outside the home was 29.7%; 18.9% for breakfast, 53.5% for lunch, and 19.6% for dinner [51]. A study conducted with an adult population in Europe (45–65 y), which preestablished 11 meal times, found that the number of meals/day was (mean ± SD) 4.3 ± 0.8 and 4.5 ± 1.0 for men and women in France, respectively, 5.2 ± 1.1 and 5.0 ± 0.9 for men and women in Norway, 5.7 ± 1.3 and 5.4 ± 1.0 for men and women in Belgium, 5.5 ± 31.3 and 5.7 ± 1.0 for men and women in the Czech Republic and 7.0 ± 1.6 and 7.1 ± 1.3 for men and women in the Netherlands. In Belgium and the Netherlands, subjects with a lower level of education were found to eat more meals/day than those with a higher educational level, and as reported here, the prevalence of the three main meals ranges from 79.8% (dinner in Norway) to 98.1% (breakfast in the Czech Republic) [20].

Comparisons with previous studies are difficult not only due to the different definitions and classifications that are made of the number of meals but also due to the methods used in the estimation of the prevalence, the frequency/day, and in the analytical approach, where the biological approaches are more common than socioeconomic approaches. In Colombia, the inability of households to physically access food measured through the ELCSA [37, 52] is one of the main determinants of alimentary and nutritional transitions [17–20, 25–29]. However, fewer meals/day, especially midmorning and midafternoon meals, do not necessarily translate into a lower quality diet if, as has been evidenced in the Colombian case, in population terms, there are no deficiencies in the consumption of energy and proteins [19, 28]. There are often such deficiencies among vulnerable groups (indigenous, displaced population, extremely poor, migrants, etc.), but this issue is beyond the scope of the current study.

Two or three meals/day, in particular the main ones and especially breakfast and in addition, fasting between 12 and 16 hours, are an ideal reported in some studies to maintain body weight, increase insulin sensitivity, decrease serum cholesterol and the sensation of hunger, improve circadian rhythm, decrease inflammation, increase resistance to stress, and improve intestinal microbiota and other risk markers for the development of cardiovascular and chronic disease in general [12]. However, the above statement is only a hypothesis that deserves further study and should be carefully observed. Based on the hours in which each of the meals that make up the pattern of meals in the Colombian population are made, the minimum fasting interval is 9 hours, and the maximum is 10 hours and 30 minutes. We established that the pattern of meals is equivalent to the pattern of type “A,” with three main meals and two or three intermediate snacks, all taken at an interval of 15 hours of the day [13]. In addition, two of them–dinner and a smaller snack–are made at night (Figure 4(s)). In Colombia, ENSIN data show that the average value of the BMI continues to increase. This shift to the right of the weight distribution has occurred under the meal pattern reported here (Type A) [13, 17].

Based on the prevalence and the place, where meals are made, it can be hypothesized that Colombia is far from being an industrialized society, where basic preindustrial and cultural relationships with food prevail over economic ones. Making the main meals mostly at home shows both a high level of economic dependence as well as limited participation in the formal production apparatus and the industry. The unemployment rate in 2015 was 8.9%, and the informal employment rate was 48.5%, thus supporting this hypothesis [53]. While in France, 53.5% of the subjects eat lunch outside the home [51], only 26.0% do so in Colombia.

### 4.1. Scope and Limitations of the Study

The main strength of this study is that the data come from a national survey, with high-quality data obtained through the 24 HR and FFQ methods. However, given the cross-sectional nature of the data, it is not possible to establish causal relationships. Another limitation is that, given the intentionality of the analysis, the number of meals/day, their prevalence or their frequency/day, or even the usual absolute intake in each type of meal (kilocalories) or their relative contribution (%) to the total energy intake (kilocalories/day) cannot be equated with the quality of the diet. This study is another piece of a complex puzzle to try to characterize different aspects of what has been conceived as the alimentary transition in Colombia. The methods used, such as the questionnaires applied in ENSIN-2015, can be used to search for causal relationships with biological variables and, in addition, to establish baselines in comparisons with future ENSINs and with studies that aim to establish the impact of food insecurity, wealth index, poverty, employment rate, income, economic or political crises, etc., and other socioeconomic variables on the number of meals/day. It is well known that the coronavirus SARS-CoV-2 pandemic and its social, economic, and political consequences have negatively impacted, first acutely and now chronically, all the indicators that potentially modify meal patterns [54, 55].

Finally, the results can be incorporated into public policy to overcome the vision focused on nutrients or foods and complement it with eating patterns and meals, closer to the daily life of the subjects, and that allow improvements in consumption habits and related practices.

## Figures and Tables

**Table 1:** The number of meals per day^*∗*^ made by the Colombian population between 3 and 4 years of age according to sociodemographic characteristics. National Survey of Nutritional Situation in Colombia (ENSIN-2015).

Variable	*n*	Mean	SE	*P* value^†^	Adjusted difference (95% CI)	*P* value^‡^
Overall	3127	4.4	0.1			

Sex				0.129		0.140
Males	1374	4.4	0.1		—	
Females	1753	4.3	0.1		−0.1 (−0.3, 0.0)	

Height-for-age *Z*-score§				0.015		0.223
<−2	236	4.8	0.1		0.1 (−0.2, 0.3)	
−2 to <−1	577	4.7	0.1		−0.1 (−0.3, 0.1)	
−1 to 1	1153	4.9	0.0		—	
>1 to 2	113	5.4	0.2		0.4 (0.1, 0.7)	
>2	20	4.6	0.5		−0.4 (−1.4, 0.6)	

BMI-for-age *Z*-score§				0.512		0.484
<−2	31	4.8	0.1		0.2 (−0.2, 0.6)	
−2 to <−1	192	4.6	0.2		−0.2 (−0.5, 0.1)	
−1 to 1	1375	4.9	0.1		—	
>1 to 2	386	4.9	0.1		−0.1 (−0.4, 0.1)	
>2	112	4.7	0.2		−0.3 (−0.6, 0.0)	

Education of head				0.011		0.002
<5 (primary or less)	749	4.3	0.1		0.0 (−0.2, 0.2)	
5 to <11	1136	4.3	0.1		—	
11 to <16	1088	4.5	0.1		0.4 (0.2, 0.6)	
≥16 (university)	143	4.6	0.3		0.4 (−0.1, 0.9)	

Wealth index, quintiles|				0.997		0.815
Q1	1677	4.3	0.1		0.0 (−0.4, 0.4)	
Q2	729	4.5	0.1		0.2 (−0.2, 0.6)	
Q3	416	4, 5	0.1		0.2 (−0.2, 0.6)	
Q4	305	4, 2	0.2		—	

Food insecurity in the home				<0.0001		<0.0001
No	1090	4.6	0.1		—	
Mild	1143	4.3	0.1		−0.2 (−0.4, −0.0)	
Moderate	510	4.2	0.1		−0.4 (−0.6, −0.1)	
Severe	384	4.0	0.1		−0.6 (−0.9, −0.3)	

Urbanicity				0.445		0.319
Big cities¶	635	4.2	0.1		—	
100.001 a 1.000.000 population	678	4.6	0.1		0.3 (−0.0, 0.5)	
0 a 100000 population	1000	4.4	0.1		0.2 (0.1, 0.5)	
Disperse population	814	4.3	0.1		0.1 (−0.2, 0.5)	

Country region				0.333		0.297
Central	633	4.6	0.1		—	
Atlantic (north)	829	4.2	0.1		−0.4 (−0.6, 0.1)	
Oriental	555	4.5	0.1		−0.1 (−0.3, 0.2)	
Pacific (west)	424	4.5	0.1		−0.1 (−0.4, 0.3)	
Bogotá	200	4.2	0.1		−0.5 (−0.8, −0.1)	
Amazonia-Orinoquia	486	4.4	0.1		−0.2 (−0.5, 0.0)	

*n*The analyzed sample may be less than 3127 due to missing values. ^*∗*^Based on FFQ. ^†^Test for linear trend for ordinal predictors. For sex, urbanicity, and country region, P is from ANOVA. All tests incorporated the complex sampling survey design. ^‡^From linear regression models with the number of meals as continuous result and indicator variables in the table as predictors except for height-for-age and BMI-for-age. The estimates for education come from a model that excludes the wealth index and food security, which could be on the causal path. The wealth index estimates excluded food security. ^§^According to the WHO [48]. | The wealth index is a composite measure of a household's cumulative living standard. The wealth index is calculated using easy-to-collect data on a household's ownership of selected assets such as televisions and bicycles, materials used for housing construction, type of water supply, and sanitation facilities. ¶ Bogotá, Barranquilla, Medellín, CAli.

**Table 2:** The number of meals per day^*∗*^ made by the Colombian population between 5 and 12 years of age according to sociodemographic characteristics. National Survey of Nutritional Situation in Colombia (ENSIN-2015).

Variable	*n*	Mean	SE	*P* value^†^	Adjusted difference	*P* value^‡^
					(95% CI)	
Overall	6274	4.5	0.0			

Sex				0.158		0.207
Males	3556	4.5	0.0		—	
Females	2718	4.4	0.1		−0.1 (−0.3, 0.1)	

Height-for-age Z-score§				0.001		0.032
<−2	466	4.6	0.1		−0.1 (−0.4, 0.1)	
−2 to <−1	1240	4.7	0.1		−0.1 (−0.3, 0.0)	
−1 to 1	2506	4.9	0.0		—	
>1 to 2	229	5.0	0.1		0.1 (−0.2, 0.3)	
>2	52	5.0	0.3		0.1 (−0.4, 0.6)	

BMI-for-age Z-score§				0.848		0.272
<−2	73	4.9	0.2		0.1 (−0.3, 0.4)	
−2 to <−1	378	4.7	0.2		−0.2 (−0.5, 0.1)	
−1 to 1	2966	4.9	0.0		—	
>1 to 2	783	4.9	0.1		0.0 (−0.2, 0.2)	
>2	279	4.7	0.1		−0.4 (−0.6, −0.2)	

Education of head				0.186		0.211
<5 (primary or less)	1662	4.4	0.1		−0.0 (−0.2, 0.2)	
5 to <11	2219	4.4	0.0		—	
11 to <16	1996	4.5	0.1		0.0 (−0.1, 0.2)	
≥16 (university)	357	4.7	0.2		0.3 (−0.2, 0.8)	

Wealth index, quintiles|				0.876		0.846
Q1	3484	4.5	0.0		0.1 (−0.2, 0.4)	
Q2	1345	4.5	0.1		0.1 (−0.2, 0.5)	
Q3	1002	4.6	0.1		0.1 (−0.2, 0.5)	
Q4	443	4.4	0.2		—	

Food insecurity in the home				<0.0001		<0.0001
No	2181	4.6	0.1		—	
Mild	2187	4.5	0.0		−0.0 (−0.2, 0.1)	
Moderate	1127	4.5	0.1		−0.0 (−0.2, 0.1)	
Severe	776	3.9	0.1		−0.6 (−1.0, −0.3)	

Urbanicity				0.224		0.865
Big cities¶	1233	4.5	0.0		—	
100.001 a 1.000.000 population	1125	4, 5	0.1		0.1 (−0.1, 0.3)	
0 a 100000 population	2562	4, 4	0.1		0.0 (−0.1, 0.2)	
Disperse population	1354	4.4	0.1		−0.0 (−0.2, 0.2)	

Country region				0.397		0.210
Central	1671	4.5	0.0		—	
Atlantic (north)	785	4.3	0.1		−0.1 (−0.3, 0.2)	
Oriental	985	4.6	0.1		0.2 (−0.0, 0.4)	
Pacific (west)	574	4.6	0.1		0.2 (−0.0, 0.4)	
Bogotá	589	4.6	0.1		0.1 (−0.1, 0.4)	
Amazonia-Orinoquia	1670	4.3	0.1		−0.1 (−0.3, 0.1)	

*n* The analyzed sample may be less than 6274 due to missing values. ^*∗*^Based on FFQ. ^†^Test for linear trend for ordinal predictors. For sex, urbanicity, and country region *P* is from ANOVA. All tests incorporated the complex sampling survey design. ^‡^From linear regression models with the number of meals as continuous result and indicator variables in the table as predictors except for height-for-age and BMI-for-age. The estimates for education come from a model that excludes the wealth index and food security, which could be on the causal path. The wealth index estimates excluded food security. ^§^According to the WHO [48]. | The wealth index is a composite measure of a household's cumulative living standard. The wealth index is calculated using easy-to-collect data on a household's ownership of selected assets such as televisions and bicycles, materials used for housing construction, type of water supply, and sanitation facilities. ¶ Bogotá, Barranquilla, Medellín, CAli.

**Table 3:** The number of meals per day^*∗*^ made by the Colombian population between 13 and 17 years of age according to sociodemographic characteristics. National Survey of Nutritional Situation in Colombia (ENSIN-2015).

Variable	*n*	Mean	SE	*P* value^†^	Adjusted difference (95% CI)	*P* value^‡^
Overall	7110	4.4	0.0			

Sex				0.114		0.142
Males	3402	4.4	0.1		—	
Females	3708	4.3	0.1		−0.1 (−0.3, 0.0)	

Height-for-age *Z*-score§				0.713		0.509
<−2	496	4.7	0.1		−0.1 (−0.3, 0.2)	
−2 to <−1	1369	4.9	0.1		0.0 (−0.1, 0.2)	
−1 to 1	2693	4.9	0.1		—	
>1 to 2	262	4.8	0.2		−0.1 (−0.5, 0.3)	
>2	69	4.6	0.2		−0.2 (−0.7, 0.2)	

BMI-for-age *Z*-score§				0.150		0.385
<−2	81	4.3	0.2		−0.4 (−0.8, −0.1)	
−2 to <−1	457	4.9	0.1		0.1 (−0.1, 0.3)	
−1 to 1	3185	4.8	0.1		—	
>1 to 2	875	4.9	0.1		0.0 (−0.2, 0.2)	
>2	293	4.9	0.1		0.1 (−0.2, 0.4)	

Education of head				<0.0001		0.001
<5 (primary or less)	2065	4.2	0.1		.0.1 (−0.3, 0.0)	
5 to <11	2402	4.3	0.1		—	
11 to <16	2252	4.5	0.1		0.1 (−0.1, 0.3)	
≥16 (university)	355	4.9	0.1		0.1 (0.2, 0.8)	

Wealth index, quintiles|				0.306		0.592
Q1	3432	4.2	0.1		−0.1 (−0.5, 0.2)	
Q2	1920	4.5	0.1		0.2 (−0.1, 0.5)	
Q3	1145	4.5	0.1		0.1 (−0.2, 0.4)	
Q4	613	4.3	0.1		—	

Food insecurity in the home				<0.0001		<0.0001
No	2388	4.5	0.1		—	
Mild	2585	4.4	0.1		−0.1 (−0.2, 0.1)	
Moderate	1282	4.3	0.1		−0.2 (−0.4, −0.0)	
Severe	855	3.8	0.1		−0.6 (−0.8, 0.4)	

Urbanicity				0.344		0.780
Big cities¶	945	4.4	0.1		—	
100.001 a 1.000.000 population	1911	4.4	0.1		−0.0 (−0.2, 0.2)	
0 a 100000 population	2514	4.5	0.1		0.1 (−0.1, 0.3)	
Disperse population	1740	4.3	0.1		−0.1 (−0.3, 0.1)	

Country region				0.857		0.290
Central	1668	4.5	0.1		—	
Atlantic (north)	1383	4.1	0.1		−0.3 (−0.5, −0.2)	
Oriental	1176	4.7	0.1		0.1 (−0.0, 0.3)	
Pacific (west)	1272	4.4	0.1		−0.2 (−0.4, −0.0)	
Bogotá	400	4.4	0.1		−0.3 (−0.6, −0.0)	
Amazonia-Orinoquia	1211	4.3	0.1		−0.1 (−0.3, −0.1)	

*n* The analyzed sample may be less than 7110 due to missing values. ^*∗*^Based on FFQ. ^†^Test for linear trend for ordinal predictors. For sex, urbanicity, and country region, *P* is from ANOVA. All tests incorporated the complex sampling survey design. ^‡^From linear regression models with the number of meals as continuous result and indicator variables in the table as predictors except for height-for-age and BMI-for-age. The estimates for education come from a model that excludes the wealth index and food security, which could be on the causal path. The wealth index estimates excluded food security. § According to the WHO [48]. | The wealth index is a composite measure of a household's cumulative living standard. The wealth index is calculated using easy-to-collect data on a household's ownership of selected assets such as televisions and bicycles, materials used for housing construction, type of water supply, and sanitation facilities. ¶ Bogotá, Barranquilla, Medellín, CAli.

**Table 4:** The number of meals per day^*∗*^ made by the Colombian population between 18 and 64 years of age (non-pregnant women) according to sociodemographic characteristics. National Survey of Nutritional Situation in Colombia (ENSIN-2015).

Variable	*n*	Mean	SE	*P* value^†^	Adjusted difference (95% CI)	*P* value^‡^
Overall	9604	4.4	0.0			

Sex				0.058		0.045
Males	4496	4.4	0.0		—	
Females	5108	4.3	0.0		−0.1 (−0.2, −0.0)	

Height				0.915		0.584
130–149	1203	4.4	0.1		0.3 (0.1, 0.5)	
150–154	982	4.2	0.1		0.1 (−0.1, 0.3)	
155–159	1277	4.3	0.1		0.2 (−0.0, 0.4)	
160–164	1100	4.1	0.1		—	
165+	1714	4.4	0.1		0.2 (−0.0, 0.4)	

BMI				<0.0001		<0.0001
<18.5	3988	4.8	0.0		0.5 (0.4, 0.6)	
18.5–24.9	3319	4.4	0.0		—	
25.0–29.9	1546	4.2	0.1		−0.2 (−0.3, −0.0)	
30+	751	4.1	0.1		−0.2 (−0.4, −0.0)	

Education of head				<0.0001		<0.0001
<5 (primary or less)	2753	4.2	0.0		−0.1 (−0.3, −0.0)	
5 to <11	3224	4.3	0.1		—	
11 to <16	3042	4.4	0.1		0.1 (−0.0, 0.2)	
≥16 (university)	515	4.7	0.1		0.3 (0.1, 0.6)	

Wealth index, quintiles§				0.481		0.972
Q1	4423	4.3	0.0		−0.0 (−0.2, 0.2)	
Q2	2215	4.5	0.1		0.1 (−0.1, 0.3)	
Q3	1859	4.4	0.1		0.1 (−0.1, 0.3)	
Q4	1107	4.4	0.1		—	

Food insecurity in the home				<0.0001		<0.0001
No	3283	4.6	0.0		—	
Mild	3398	4.4	0.0		−0.2 (−0.3, −0.0)	
Moderate	1806	4.1	0.1		−0.4 (−0.7, −0.1)	
Severe	1116	4.0	0.1		−0.5 (−0.7, −0.3)	

Urbanicity				0.509		0.834
Big cities|	1011	43	0.1		—	
100.001 a 1.000.000 population	2485	4.5	0.0		0.2 (−0.0, 0.3)	
0 a 100000 population	3609	4.3	0.1		0.0 (−0.2, 0.2)	
Disperse population	2499	4.3	0.1		0.0 (−0.2, 0.3)	

Country region				0.721		0.274
Central	2280	4.4	0.0		—	
Atlantic (north)	2013	4.2	0.1		−0.2 (−0.3, −0.0)	
Oriental	1844	4.6	0.1		0.1 (−0.0, 0.3)	
Pacific (west)	1205	4.3	0.1		−0.1 (−0.3, 0.1)	
Bogotá	624	4.3	0.1		−0.2 (−0.5, 0.0)	
Amazonia-Orinoquia	1638	4.3	0.1		−0.1 (−0.3, 0.0)	

*n* The analyzed sample may be less than 9604 due to missing values. ^*∗*^Based on FFQ. ^†^Test for linear trend for ordinal predictors. For sex, urbanicity, and country region *P* is from ANOVA. All tests incorporated the complex sampling survey design. ^‡^From linear regression models with the number of meals as continuous result and indicator variables in the table as predictors except for height and BMI. The estimates for education come from a model that excludes the wealth index and food security, which could be on the causal path. The wealth index estimates excluded food security. ^§^The wealth index is a composite measure of a household's cumulative living standard. The wealth index is calculated using easy-to-collect data on a household's ownership of selected assets such as televisions and bicycles, materials used for housing construction, type of water supply, and sanitation facilities. | Bogotá, Barranquilla, Medellín, CAli.

**Table 5:** Prevalence (%), frequency/day (times per day), average kilocalories, and relative energy contribution (%) by type of meals made in the Colombian population (3 to 64 years, non-pregnant women). National Survey of Nutritional Situation in Colombia (ENSIN-2015).

Type of meals	3–4 y [3127]	5–12 y [6274]	13–17 y [7110]	18–64 y [9604]
	Estimator (95% CI)			
Total kilocalories/day^*∗*^	1699 (1543, 1854)	1822 (1626, 2018)	2304 (2210, 2398)	1993 (1948, 2038)

Before breakfast				
Prevalence (%)^†^	24.0 (19.6, 29.1)	30.2 (25.4, 35.4)	22.3 (19.7, 25, 2)	24.0 (22.1, 26.2)
Frequency/day^†^	0.23 (0.18, 0.28)	0.27 (0.22, 0.31)	0.20 (0.17, 0.23)	0.22 (0.20, 0.24)
Energy (kilocalories)^*∗*^	215 (113, 318)	149 (131, 167)	132 (88, 175)	107 (89, 124)
Relative contribution (%)^*∗*^	3.6 (1.3, 5.9)	0.7 (0.3, 1.2)	1.1 (0.5, 1.8)	1.4 (1.2, 1.6)

Breakfast				
Prevalence (%)	93.0 (89.6, 95.4)	95.7 (94.2, 96.7)	95.8 (94.3, 96.7)	96.1 (95.3, 96.8)
Frequency/day	0.93 (0.90, 0.97)	0.93 (0.90, 0.96)	0.96 (0.94, 0.98)	0.97 (0.95, 0.98)
Energy (kilocalories)	342 (292, 393)	447 (424, 470)	556 (525, 588)	491 (469, 514)
Relative contribution (%)	21.0 (18.8, 23.3)	25.1 (21.6, 28.6)	21.1 (19.9, 22.3)	22.8 (21.9, 23.6)

Mid-morning				
Prevalence (%)	47.8 (0.43, 0.52)	46.6 (41.0, 52.2)	46.8 (43.8, 49.8)	46.7 (44.1, 49.4)
Frequency/day	0.37 (0.34, 0.41)	0.38 (0.33, 0.43)	0.39 (0.36, 0.41)	0.39 (0.37, 0.42)
Energy (kilocalories)	206 (162, 251)	285 (264, 306)	357 (312, 403)	239 (218, 261)
Relative contribution (%)	7.9 (5.1, 10.7)	6.2 (2.8, 9.6)	6.0 (4.2, 7.8)	4.3 (3.8, 4.7)

Lunch				
Prevalence (%)	98.6 (97.6, 99.2)	98.8 (97.7, 99.4)	99.4 (99.0, 99.6)	98.0 (96.9, 98.8)
Frequency/day	1.0 (1.0, 1.0)	1.0 (0.98, 1.02)	1.03 (1.01, 1.04)	1.0 (1.0, 1.0)
Energy (kilocalories)	505 (412, 599)	607 (567, 646)	809 (749, 868)	727 (705, 748)
Relative contribution (%)	30.7 (26.2, 35.2)	34.2 (30.8, 37.6)	32.9 (31.0, 34.9)	35.6 (34.5, 36.7)

Mid-afternoon				
Prevalence (%)	50.8 (46.5, 55.1)	54.5 (51.2, 57.8)	52.2 (49.1, 55.4)	50.2 (47.4, 0.53)
Frequency/day	0.41 (0.37, 0.45)	0.44 (0.39, 0.48)	0.42 (0.40, 0.45)	0.40 (0.38, 0.43)
Energy (kilocalories)	300 (268, 331)	299 (274, 324)	395 (348, 443)	299 (270, 327)
Relative contribution (%)	13.5 (10.7, 16.3)	7.5 (3.4, 11.7)	9.6 (6.8, 12.4)	8.1 (7.2, 9.0)

Dinner				
Prevalence (%)	94.5 (90.7, 96.8)	95.5 (92.7, 97.3)	94.4 (92.1, 96.1)	94.6 (93.0, 95.9)
Frequency/day	0.95 (0.92, 0.96)	0.93 (0.91, 0.96)	0.95 (0.92, 0.97)	0.96 (0.94, 0.97)
Energy (kilocalories)	367 (319, 415)	457 (399, 515)	634 (576, 693)	576 (549, 603)
Relative contribution (%)	21.1 (19.5, 22.8)	24.7 (22.5, 26.9)	25.3 (23.6, 27.0)	25.4 (24.1, 26.7)

After dinner				
Prevalence (%)	26.5 (20.6, 33.2)	25.2 (30.0, 31.2)	25.2 (21.1, 20.7)	25.1 (22.9, 27.4)
Frequency/day	0.20 (0.15, 0.25)	0.17 (0.13, 0.22)	0.18 (0.15, 0.21)	0.18 (0.16, 0.19)
Energy (kilocalories)	155 (134, 175)	206 (182, 230)	311 (240, 382)	260 (222, 297)
Relative contribution (%)	2.2 (1.3, 3.1)	1.5 (0.7, 2.4)	4.0 (1.2, 6.8)	2.4 (2.1, 2.9)

^*∗*^Based on 24-hour dietary recall. 1 kcal/d = 4.18 kJ/d; Based on usual intake, incorporating intra-subject variability [45]. ^†^Based on FFQ.

**Table 6:** Places where the main meals are made in the Colombian population (3 to 64 years, non-pregnant women). National Survey of Nutritional Situation in Colombia (ENSIN-2015).

Type of food Place where it takes place^*∗*^	3–4 y [3127]	5–12 y [6274]	13–17 y [7110]	18–64 y [9604]
	Prevalence (95% CI)			
Before breakfast				
Home	96.0 (93.2, 97.6)	93.9 (91.3, 95.7)	94.6 (91.8, 96.5)	93.0 (90.0, 95.2)

Breakfast				
Home	87.2 (84.2, 89.7)	85.8 (81.0, 89.5)	87.1 (84.9, 88.9)	87.6 (86.2, 88.9)
College/School/University	3.3(2.3, 4.9)	3.5 (2.8, 4.4)	3.3 (2.6, 4.0)	3.0 (2.4, 3.7)
Work	7.1 (5.2, 9.5)	8.7 (5.6, 13.1)	7.2 (5.9, 8.9)	7.0 (6.1, 8.2)
Family home or friend	1.0 (0.5, 1.9)	0.8 (0.5, 1.3)	0.7 (0.5, 1.1)	0.8 (0.6, 1.2)
Coffee shop	0.0 (0.0, 0.0)	0.1 (0.0, 0.5)	0.3 (0.1, 0.7)	0.3 (0.1, 0.6)
On the street (street stall)	0.5 (0.2, 1.4)	0.2 (0.1, 0.4)	0.3 (0.1, 7.4)	0.5 (0.3, 0.8)
Restaurant	0.5 (0.2, 1.1)	0.6 (0.3, 1.0)	0.6 (0.3, 1.0	0.3 (0.2, 0.5)

Mid-morning				
Home	42.2 (37.1, 47.5)	41.1 (37.8, 44.6)	44.7 (39.0, 50.6)	41.9 (38.5, 45.3)
College/School/University	32.2 (27.3, 37.5)	34.9 (30.0, 40.2)	31.3 (27.1 35.8)	29.5 (26.7, 32.4)
Work	21.1 (15.7, 27.8)	17.6 (13.5, 22.7)	18.9 (15.1, 23.4)	22.3 (19.4, 25.5)
On the street (street stall)	1.6 (0.9, 2.7)	0.9 (0.5, 1.7)	1.4 (0.8, 2.4)	1.7 (1.0, 2.7)
Coffee shop	1.1 (0.5, 2.4)	1.4 (0.7, 2.5)	1.5 (0.8, 2.8)	1.2 (0.8, 1.9)
Family home or friend	0.6 (0.3, 1.3)	2.3 (0.8, 6.8)	0.6 (0.4, 1.0)	0.9 (0.6, 1.4)

Lunch				
Home	74.5 (70.3, 78.2)	71.5 (65.4, 76.9)	74.8 (72.3, 77.0)	75.0 (72.6, 77.3)
Work	15.1 (11.7, 19.2)	15.6 (10.3, 23.0)	13.5 (11.5, 15.9)	12.2 (10.7, 13.9)
College/School/University	5.9 (4.2, 8.1)	7.2 (5.7, 9.0)	6.1 (4.8, 7.7)	6.0 (5.0, 7.3)
Restaurant	2.6 (1.7, 3.8)	2.0 (1.5, 2.7)	3.0 (2.2, 4.1)	3.8 (2.4, 6.1)
Family home or friend	1.1 (0.7, 1.7)	2.0 (1.0, 3.9)	1.5 (1.0, 2.1)	1.7 (1.3, 2.3)
Institutional program	0.5 (0.2, 1.0)	0.6 (0.4, 1.1)	0.6 (0.3, 1.4)	0.6 (0.4, 0.9)

Mid-afternoon				
Home	72.3 (67.4, 76.8)	67.6 (63.1, 71.8)	69.8 (66.6, 72.9)	67.2 (64.2, 70.2)
Work	11.4 (8.2, 15.7)	14.4 (10.3, 19.1)	12.2 (9.8, 15.2)	14.2 (11.9, 16.7)
College/School/University	11.0 (9.2, 13.2)	11.1 (9.1, 13.6)	11.8 (10.0, 14.0)	12.2 (10.3, 14.4)
On the street (street stall)	2.4 (1.1, 5.3)	2.0 (1.1, 3.3)	1.2 (0.7, 1.8)	1.7 (1.2, 2.5)
Family home or friend	0.6 (0.3, 1.2)	1.7 (1.0, 3.0)	2.4 (1.7, 3.3)	1.5 (1.1, 2.3)
Coffee shop	1.0 (0.5, 2.0)	0.9 (0.5, 1.5)	1.2 (0.7, 2.0)	1.8 (1.0, 3.3)

Dinner				
Home	96.9 (95.8, 97.8)	96.2 (94.7, 97.3)	96.5 (95.8, 97.2)	95.8 (94.8, 96.6)
College/School/University	0.2 (0.1, 0.6)	0.5 (0.2, 1.3)	0.4 (0.2, 0.7)	0.7 (0.3, 1.6)
Family home or friend	0.7 (0.3, 1.4)	1.2 (0.5, 3.1)	0.7 (0.4, 1.0)	1.0 (0.7, 1.5)
Work	1.5 (0.8, 2.7)	1.3 (0.9, 2.0)	1.9 (1.4, 2.6)	1.6 (1.2, 2.2)

After dinner				
Home	94.8 (92.6, 96.3)	93.5 (91.2, 95.2)	93.3 (90.9, 95.1)	93.7 (91.3, 95.5)
Work	1.4 (0.5, 3.7)	3.4 (1.8, 6.5)	1.2 (0.6, 2.2)	0.8 (0.4, 1.8)
On the street (street stall)	3.1 (1.7, 5.4)	1.2 (0.7, 2.1)	2.9 (1.7, 4.9)	3.2 (1.8, 5.3)
Family home or friend	0.2 (0.0, 0.5)	1.1 (0.5, 2.3)	0.6 (0.3, 1.2)	0.5 (0.3, 1.0)

^*∗*^Based on FFQ.

## Data Availability

To access the ENSIN 2015 public database, you must register in the repository of the Ministry of Public Health: repositorio@minsalud.gov.co and make the request through the format available at: https://www.minsalud.gov.co/sites/rid/paginas/freesearchresultsf.aspx?k=Base%20de%20datos%20Encuesta%20Nacional%20de%20la%20Situaci%C3%B3n%20Nutricional%20ENSIN%202015.

## Additional Points

The lead author affirms that this manuscript is an honest, accurate, and transparent account of the study being reported. The reporting of this work is compliant with STROBE guidelines. The lead author affirms that no important aspects of the study have been omitted and that any discrepancies from the study as planned have been explained.

## Supplementary Materials

Supplementary Materials Figure 1(s): Number of meals per day (mean and 95% CI) according to age in the Colombian population (3–64 y, non-pregnant women). National Survey of Nutritional Situation in Colombia (ENSIN-2015). Figure 2(s): Probability of meal type per day according to food security at the home in the Colombian population (3–64 y, non-pregnant women). National Survey of Nutritional Situation in Colombia (ENSIN-2015). Figure 3(s): Relative contribution (%) to total kilocalories/day according to the type of meal in the Colombian population (3–64 y, non-pregnant women). National Survey of Nutritional Situation in Colombia (ENSIN-2015). Figure 4(s): Time of day in which meal times are made (Median, P_25_, P_75_) and density by the number of foods in them in the Colombian population (3–64 y, non-pregnant women). National Survey of Nutritional Situation in Colombia (ENSIN-2015). Appendix. Appendix 1-(a) to Appendix 1-(g) present the contributing food categories for each type of meal (proportion, %). Due to the volume of presenting this information in detail by subcategories that are part of each category or foods that make up each subcategory, this can be requested from the authors with the intention of collaborative work. There are four supplementary figures and one appendix.
