# Supplementary material for: Meal Pattern in the Colombian Population: Results of the National Nutrition Survey. ENSIN, 2015
Source: J Nutr Metab. 2022 Aug 25;2022:1047524. doi: 10.1155/2022/1047524 (PMC9436622; doi:10.1155/2022/1047524)
Supplement: Supplementary Materials — Figure 1(s): Number of meals per day (mean and 95% CI) according to age in the Colombian population (3–64 y, non-pregnant women). National Survey of Nutritional Situation in Colombia (ENSIN-2015). Figure 2(s): Probability of meal type per day according to food security at the home in the Colombian population (3–64 y, non-pregnant women). National Survey of Nutritional Situation in Colombia (ENSIN-2015). Figure 3(s): Relative contribution (%) to total kilocalories/day according to the type of meal in the Colombian population (3–64 y, non-pregnant women). National Survey of Nutritional Situation in Colombia (ENSIN-2015). Figure 4(s): Time of day in which meal times are made (Median, P25, P75) and density by the number of foods in them in the Colombian population (3–64 y, non-pregnant women). National Survey of Nutritional Situation in Colombia (ENSIN-2015). Appendix. Appendix 1-(a) to Appendix 1-(g) present the contributing food categories for each type of meal (proportion, %). Due to the volume of presenting this information in detail by subcategories that are part of each category or foods that make up each subcategory, this can be requested from the authors with the intention of collaborative work. There are four supplementary figures and one appendix. [file 1047524.f1.docx]

**Meal pattern in the Colombian population: results of the National Nutrition Survey.**

**ENSIN, 2015**

María del Pilar Zea**^1^**, Oscar F. Herrán**^2^**

^1^ Pontificia Universidad Javeriana Seccional Cali, Facultad de Ciencias de la Salud, Departamento de Alimentación y Nutrición. Calle 18 N 118-250, Cali, Valle del Cauca, Colombia. ^2^ Escuela de Nutrición y Dietética, Universidad Industrial de Santander, Carrera 32 No. 29-31, Bucaramanga, Santander, Colombia. [herran@uis.edu.co](mailto:herran@uis.edu.co)

Online Supplementary Material

|  | Page |
| --- | --- |
| **Figure 1S** Number of meals per day (mean and 95% CI) according to age in the Colombian population (3-64y, *non-pregnant women*). National Survey of Nutritional Situation in Colombia (ENSIN-2015). | 3 |
| **Figure 2S** Probability of meal type per day according to food security at the home in the Colombian population (3-64 y, *non-pregnant women*). National Survey of Nutritional Situation in Colombia (ENSIN-2015). | 4 |
| **Figure 3S** Relative contribution (%) to total kilocalories / day according to type of meal in the Colombian population (3-64 y, *non-pregnant women*). National Survey of Nutritional Situation in Colombia (ENSIN-2015). | 5 |
| **Figure 4S** Time of day in which meal times are made (Median, P_25_, P_75_) and density by the number of foods in them in the Colombian population (3-64 y, *non-pregnant women*). National Survey of Nutritional Situation in Colombia (ENSIN-2015). | 6 |
|  |  |
| **APPENDIX** |  |
|  |  |
| 1-a Food categories present in the “before breakfast” meal, (3-64 y, *non-pregnant women*). National Survey of Nutritional Situation in Colombia (ENSIN-2015). | 7 |
| 1-b Food categories present in the “breakfast” meal, (3-64 y, *non-pregnant women*). National Survey of Nutritional Situation in Colombia (ENSIN-2015). | 8 |
| 1-c Food categories present in the “Mid-morning” meal, (3-64 y, *non-pregnant women*). National Survey of Nutritional Situation in Colombia (ENSIN-2015). | 9 |
| 1-d Food categories present in the “Lunch” meal, (3-64 y, *non-pregnant women*). National Survey of Nutritional Situation in Colombia (ENSIN-2015). | 10 |
| 1-e Food categories present in the “Mid-afternoon” meal, (3-64 y, *non-pregnant women*). National Survey of Nutritional Situation in Colombia (ENSIN-2015). | 11 |
| 1-f Food categories present in the “Dinner” meal, (3-64 y, *non-pregnant women*). National Survey of Nutritional Situation in Colombia (ENSIN-2015). | 12 |
| 1-g Food categories present in the “After dinner” meal, (3-64 y, *non-pregnant women*). National Survey of Nutritional Situation in Colombia (ENSIN-2015). | 13 |

**Figure 1S** Number of meals per day (mean and 95% CI) according to age in the Colombian population (3-64y, *non-pregnant women*). National Survey of Nutritional Situation in Colombia (ENSIN-2015).

**Figure 2S** Probability of meal type per day according to food security at the home in the Colombian population (3-64 y, *non-pregnant women*). National Survey of Nutritional Situation in Colombia (ENSIN-2015).

**Figure 3S** Relative contribution (%) to total kilocalories / day according to type of meal in the Colombian population (3-64 y, *non-pregnant women*). National Survey of Nutritional Situation in Colombia (ENSIN-2015).

**Figure 4S** Time of day in which meal times are made (Median, P_25_, P_75_) and density by the number of foods in them in the Colombian population (3-64 y, *non-pregnant women*). National Survey of Nutritional Situation in Colombia (ENSIN-2015).

**APPENDIX**

**Appendix 1-a**

Food categories present in the “before breakfast” meal, (3-64 y, *non-pregnant women*). National Survey of Nutritional Situation in Colombia (ENSIN-2015).

**Before Breakfast**

|  | **Proportion** | **Order** |
| --- | --- | --- |
|  | **%** |  |
|  |  |  |
| Packaged foods (industrialized snacks...) | 1.9 | 9 |
| Baby food | 3.2 | 8 |
| Sugars | 21.9 | 2 |
| Alcoholic drinks | 0.0 | 12 |
| Non-alcoholic beverages | 24.4 | 1 |
| Meats, eggs, dried legumes, nuts and seeds | 1.2 | 9 |
| Cereals and cereal derivatives | 13.5 | 4 |
| Condiments, sauces and dehydrated soups | 0.0 | 12 |
| Sweets and desserts | 1.2 | 9 |
| Fruits and vegetables | 12.2 | 5 |
| Fats | 1.0 | 10 |
| Milk and derivatives | 7.3 | 6 |
| Others | 14.9 | 3 |
| Preparations | 0.0 | 12 |
| Roots, tubers and bananas | 0.4 | 11 |
| Supplements and nutritional supplements | 1.2 | 9 |

**Note**: The proportion (%) was established based on the number of records made in the of the last 24 hours recall [*n*=617,880]. The accumulated frequency can be greater than 100, due to the approximations to the next decimal.

**Appendix 1-b**

Food categories present in the “breakfast” meal, (3-64 y, *non-pregnant women*). National Survey of Nutritional Situation in Colombia (ENSIN-2015).

**Breakfast**

|  | **Proportion** | **Order** |
| --- | --- | --- |
|  | **%** |  |
|  |  |  |
| Packaged foods (industrialized snacks...) | 0.0 | 12 |
| Baby food | 0.0 | 12 |
| Sugars | 12.7 | 4 |
| Alcoholic drinks | 0.0 | 12 |
| Non-alcoholic beverages | 8.8 | 6 |
| Meats, eggs, dried legumes, nuts and seeds | 14.7 | 2 |
| Cereals and cereal derivatives | 22.0 | 1 |
| Condiments, sauces and dehydrated soups | 1.0 | 11 |
| Sweets and desserts | 2.4 | 9 |
| Fruits and vegetables | 7.8 | 7 |
| Fats | 9.1 | 5 |
| Milk and derivatives | 14.4 | 3 |
| Others | 3.1 | 9 |
| Preparations | 0.0 | 12 |
| Roots, tubers and bananas | 5.8 | 8 |
| Supplements and nutritional supplements | 2.6 | 10 |

**Note**: The proportion (%) was established based on the number of records made in the of the last 24 hours recall [*n*=617,880]. The accumulated frequency can be greater than 100, due to the approximations to the next decimal.

**Appendix 1-c**

Food categories present in the “Mid-morning” meal, (3-64 y, *non-pregnant women*). National Survey of Nutritional Situation in Colombia (ENSIN-2015).

**Mid-morning**

|  | **Proportion** | **Order** |
| --- | --- | --- |
|  | **%** |  |
|  |  |  |
| Packaged foods (industrialized snacks...) | 4.2 | 8 |
| Baby food | 0.2 | 14 |
| Sugars | 6.9 | 6 |
| Alcoholic drinks | 1.0 | 11 |
| Non-alcoholic beverages | 17.5 | 3 |
| Meats, eggs, dried legumes, nuts and seeds | 6.1 | 7 |
| Cereals and cereal derivatives | 19.0 | 1 |
| Condiments, sauces and dehydrated soups | 0.1 | 15 |
| Sweets and desserts | 7.7 | 5 |
| Fruits and vegetables | 17.8 | 2 |
| Fats | 1.9 | 10 |
| Milk and derivatives | 11.0 | 4 |
| Others | 2.8 | 9 |
| Preparations | 0.4 | 13 |
| Roots, tubers and bananas | 1.9 | 10 |
| Supplements and nutritional supplements | 0.8 | 12 |

**Note**: The proportion (%) was established based on the number of records made in the of the last 24 hours recall [*n*=617,880]. The accumulated frequency can be greater than 100, due to the approximations to the next decimal.

**Appendix 1-d**

Food categories present in the “Lunch” meal, (3-64 y, *non-pregnant women*). National Survey of Nutritional Situation in Colombia (ENSIN-2015).

**Lunch**

|  | **Proportion** | **Order** |
| --- | --- | --- |
|  | **%** |  |
|  |  |  |
| Packaged foods (industrialized snacks...) | 0.0 | 12 |
| Baby food | 0.0 | 12 |
| Sugars | 3.7 | 7 |
| Alcoholic drinks | 0.0 | 12 |
| Non-alcoholic beverages | 5.9 | 6 |
| Meats, eggs, dried legumes, nuts and seeds | 16.6 | 2 |
| Cereals and cereal derivatives | 13.9 | 4 |
| Condiments, sauces and dehydrated soups | 2.8 | 8 |
| Sweets and desserts | 0.1 | 11 |
| Fruits and vegetables | 29.9 | 1 |
| Fats | 9.7 | 5 |
| Milk and derivatives | 1.5 | 9 |
| Others | 0.0 | 12 |
| Preparations | 1.0 | 10 |
| Roots, tubers and bananas | 14.2 | 3 |
| Supplements and nutritional supplements | 0.0 | 12 |

**Note**: The proportion (%) was established based on the number of records made in the of the last 24 hours recall [*n*=617,880]. The accumulated frequency can be greater than 100, due to the approximations to the next decimal.

**Appendix 1-e**

Food categories present in the “Mid-afternoon” meal, (3-64 y, *non-pregnant women*). National Survey of Nutritional Situation in Colombia (ENSIN-2015).

**Mid-afternoon**

|  | **Proportion** | **Order** |
| --- | --- | --- |
|  | **%** |  |
|  |  |  |
| Packaged foods (industrialized snacks...) | 3.9 | 8 |
| Baby food | 0.0 | 14 |
| Sugars | 7.9 | 6 |
| Alcoholic drinks | 1.7 | 11 |
| Non-alcoholic beverages | 18.0 | 2 |
| Meats, eggs, dried legumes, nuts and seeds | 4.9 | 7 |
| Cereals and cereal derivatives | 19.7 | 1 |
| Condiments, sauces and dehydrated soups | 1.0 | 13 |
| Sweets and desserts | 12.6 | 3 |
| Fruits and vegetables | 12.1 | 4 |
| Fats | 2.1 | 10 |
| Milk and derivatives | 10.6 | 5 |
| Others | 3.2 | 9 |
| Preparations | 0.0 | 14 |
| Roots, tubers and bananas | 1.5 | 12 |
| Supplements and nutritional supplements | 0.0 | 14 |

**Note**: The proportion (%) was established based on the number of records made in the of the last 24 hours recall [*n*=617,880]. The accumulated frequency can be greater than 100, due to the approximations to the next decimal.

**Appendix 1-f**

Food categories present in the “Dinner” meal, (3-64 y, *non-pregnant women*). National Survey of Nutritional Situation in Colombia (ENSIN-2015).

**Dinner**

|  | **Proportion** | **Order** |
| --- | --- | --- |
|  | **%** |  |
|  |  |  |
| Packaged foods (industrialized snacks...) | 0.0 | 11 |
| Baby food | 0.0 | 11 |
| Sugars | 5.9 | 7 |
| Alcoholic drinks | 0.0 | 11 |
| Non-alcoholic beverages | 7.9 | 6 |
| Meats, eggs, dried legumes, nuts and seeds | 17.0 | 3 |
| Cereals and cereal derivatives | 18.1 | 2 |
| Condiments, sauces and dehydrated soups | 3.0 | 9 |
| Sweets and desserts | 1.0 | 10 |
| Fruits and vegetables | 19.2 | 1 |
| Fats | 10.4 | 4 |
| Milk and derivatives | 5.8 | 8 |
| Others | 1.0 | 10 |
| Preparations | 1.0 | 10 |
| Roots, tubers and bananas | 9.7 | 5 |
| Supplements and nutritional supplements | 0.0 | 11 |

**Note**: The proportion (%) was established based on the number of records made in the of the last 24 hours recall [*n*=617,880]. The accumulated frequency can be greater than 100, due to the approximations to the next decimal.

**Appendix 1-g**

Food categories present in the “After dinner” meal, (3-64 y, *non-pregnant women*). National Survey of Nutritional Situation in Colombia (ENSIN-2015).

**After dinner**

|  | **Proportion** | **Order** |
| --- | --- | --- |
|  | **%** |  |
|  |  |  |
| Packaged foods (industrialized snacks...) | 3.6 | 9 |
| Baby food | 0.0 | 15 |
| Sugars | 9.1 | 6 |
| Alcoholic drinks | 5.2 | 8 |
| Non-alcoholic beverages | 16.9 | 1 |
| Meats, eggs, dried legumes, nuts and seeds | 6.4 | 7 |
| Cereals and cereal derivatives | 16.5 | 2 |
| Condiments, sauces and dehydrated soups | 1.8 | 12 |
| Sweets and desserts | 9.5 | 5 |
| Fruits and vegetables | 11.0 | 3 |
| Fats | 2.7 | 10 |
| Milk and derivatives | 10.8 | 4 |
| Others | 2.6 | 11 |
| Preparations | 0.0 | 15 |
| Roots, tubers and bananas | 1.7 | 13 |
| Supplements and nutritional supplements | 1.3 | 14 |

**Note**: The proportion (%) was established based on the number of records made in the of the last 24 hours recall [*n*=617,880]. The accumulated frequency can be greater than 100, due to the approximations to the next decimal.
